# A Novel Preclinical Rat Model of Acute Compartment Syndrome

**DOI:** 10.1002/jor.70262

**Published:** 2026-08-23

**Authors:** Sishu Guan, Xingchen Lu, Chang Liu, Yi Zhang, Yang Li, Hui Zhao, Lianyang Zhang

**Affiliations:** ^1^ War Trauma Medical Center, State Key Laboratory of Trauma and Chemical Poisoning, Daping Hospital Army Medical University (Third Military Medical University) Chongqing China; ^2^ Emergency Department The General Hospital of Western Theater Command Chengdu China; ^3^ Department of Orthopaedics, Daping Hospital Army Medical University (Third Military Medical University) Chongqing China; ^4^ Department of Emergency, Daping Hospital Army Medical University (Third Military Medical University) Chongqing China; ^5^ Department of Intensive Care Unit, Daping Hospital Army Medical University (Third Military Medical University) Chongqing China; ^6^ Institute for Traffic Medicine, Daping Hospital Army Medical University (Third Military Medical University) Chongqing China

**Keywords:** acute compartment syndrome, animal model, blood perfusion, intracompartmental pressure

## Abstract

Acute compartment syndrome (ACS) is common in trauma, challenging early diagnosis and treatment. An accurate ACS model is vital for its management. Most prior animal models have induced intrafascial hypertension by exogenous interventions, focusing on the study of secondary injury post‐ACS formation. A novel rat model with spontaneous ACS formation after trauma was constructed, emphasizing early onset and development. The 300‐mmHg pressure simulated by a blood pressure cuff was applied to rat hindlimbs for 3 h. A balloon catheter connected to a manometer was developed, and intracompartmental pressure (ICP) and blood perfusion were measured continuously for 2 h. H&E staining and serum index were detected at 0, 1‐, 2‐, 3‐, and 8‐h postinjury. The results showed injured hindlimbs swelled, with ICP rising continuously, reaching 30 mmHg in 35 min, peaking at 60–80 min before entering a plateau period, effectively reproducing ACS pressure evolution. The blood perfusion showed a three‐step change, negatively correlating with ICP > 30 mmHg. Muscle tissues presented typical time‐dependent pathological changes, with progressive myofibrillar rupture, inflammatory cell infiltration, and increases in serum IL‐1β, TNF‐α, CK, and BUN over time. This study successfully established a new preclinical ACS‐like rat model. Compared with previous studies, the ICP in the hindlimbs in this study was spontaneously elevated after crush, leading to secondary injury. Furthermore, a new manometric catheter device was developed to enable continuous monitoring of ICP without repeated fluid injections. The constructed animal model is closer to the clinic, and it lays a solid foundation for ACS pathogenesis.

**Abbreviations**
ACS: acute compartment syndrome
BUN: urea nitrogen
CK: creatine kinase
ELISA: enzyme‐linked immunosorbent assay
H&E: hematoxylin and eosin
ICP: intracompartmental pressure
IL‐1β: interleukin 1β
MDA: malondialdehyde
ROI: region of interest
TNF‐α: tumor necrosis factor α

## 1 Introduction

Acute compartment syndrome (ACS) is a condition of elevated pressure within the fascia‐limited compartments of a limb where microcirculation and tissue perfusion are compromised [1]. The disease is rare, with an estimated incidence of 7.3 per 100,000 in males and 0.7 per 100,000 in females [2]. The true incidence of ACS has not been determined because most clinical studies have used fasciotomy as a diagnostic criterion [1]. ACS usually occurs secondary to physical trauma, such as bone fracture or crush injury, but may also develop during ischemia (e.g., prolonged tourniquet use), eschar contracture after extensive burns, or muscle overexertion due to strenuous exercise [3, 4, 5]. Current understanding suggests that the rapid progression of ACS results from positive feedback within the compartment, in which initial trauma or hypoxia induces tissue damage, stimulating local oxidative and inflammatory responses and increasing vascular permeability [1, 6]. Fluid leaking into the compartment increases interstitial pressure and compression of venous drainage, further increasing tissue pressure [7]. Eventually, tissue pressure approaches average arterial pressure, thereby restricting tissue perfusion and increasing hypoxic injury [8]. As the cycle repeats, increased pressure, reduced tissue perfusion, and hypoxic injury progress, culminating in extensive tissue damage and necrosis, leading to loss of muscle, nervous, and vascular tissues within the compartment [9]. The final result is often disability or loss of the limb, and in particularly severe cases, renal failure due to rhabdomyolysis, and possible death. Clinical diagnosis relies on the typical presentation of the 5Ps (Pain, Pallor, Paresthesia, Paralysis, Pulselessness) [10]. However, in clinical practice, these symptoms are poor predictors, and when present, they are terminal, losing the opportunity for surgical neuromuscular salvage [11].

Various types of animal models have been developed in the past, with dogs, pigs, rats, and turkeys as commonly used species, and the construction methods differ [12, 13, 14]. For example, the intrafascial fluid infusion method is more commonly used to simulate the pathology of high intracompartmental pressure (ICP) after trauma by continuous dripping of fluid in the intrafascial compartment [15]. The direct increase of ICP by external devices to simulate clinically high ICP is also one of the classic modeling approaches, with catheterized balloons placed within the intrafascial compartment or with an inflatable cuff around the limb [16, 17, 18]. However, these models have certain shortcomings. On the one hand, the reliance on tourniquets or vascular clamps to restrict arterial supply for creating ischemic injury and the need for surgical removal of blood vessels to eliminate collateral circulation to the compartment increase surgical complexity. On the other hand, the inflammatory reperfusion injury triggered by the sudden restoration of blood flow after removal of the tourniquet or vascular clamp is different from the diffuse focal ischemia commonly seen in limb trauma [19]. Some studies have attempted to simulate ACS by continuously infusing fluids into muscle areas to artificially increase interstitial pressure and swelling [15, 20]. While diffuse injury does occur, large volumes of fluid are reabsorbed into the circulation, again raising questions of systemic effects, which in turn limit their suitability and accuracy for simulating ACS in humans.

In past ICP measurements in animal studies, the Whitesides method or its modifications were used to obtain pressure data by injecting approximately 0.3–0.5 mL of fluid into the fascial compartment in discontinuous injections [21]. However, due to the rapid absorption of the injected fluid into the tissue, multiple measurements must be averaged, and continuous measurements are not possible, leading to large measurement errors. Multiple discontinuous injections of saline can also affect pathophysiologic processes within the tissue. Notably, previous models have mostly focused on the pathophysiologic changes following ACS formation, whereas the process of ACS formation following limb trauma has been less studied. An in‐depth understanding of this process is of great value for early intervention in ACS, reducing surgical rates, and improving long‐term prognosis.

In this study, we first developed a novel ACS injury model using limb crush to simulate the natural progression of ACS following trauma, thereby filling a major gap in preclinical research. Meanwhile, we continuously monitored ICP using a balloon catheter that did not require repeated fluid injections. Compared with previous studies, our model reproduces the development of clinical ACS and provides a physiologically relevant platform for studying the dynamic physiological changes during ACS formation.

## 2 Materials and Methods

### 2.1 Animals

All experimental animals were obtained from the experimental center of Daping Hospital, Army Military Medical University (Third Military Medical University). A total of 48 10‐week‐old male Sprague–Dawley rats weighing 250 ± 20 g were used in this study. Before the experiment, the rats were housed in an environment with suitable temperature (22 ± 2°C) and relative humidity (50 ± 10%) for 1 week of acclimatization, with free access to food and water. This study was approved by the Laboratory Animal Welfare and Ethics Committee of the Army Medical University (Approval No. SYXK (YU) 2022‐0018).

### 2.2 Construction of the ACS Model

The sample size of 48 rats was determined by a power analysis based on our pilot study (expected effect size = 0.8, *α* = 0.05, and power = 0.8), resulting in eight rats per group. The rats were randomly divided into six groups using a computer‐generated random number sequence: Sham, 0, 1, 2, 3, and 8 h. All behavioral and histological assessments were performed by an investigator blinded to group allocation. There were no exclusions. The rats were anesthetized with exhalation isoflurane (2%, 1.5 L/min) throughout the modeling procedure, and fixed in the supine position on the operating table. For the injury groups, a neonatal blood pressure cuff (Tempa‐Kuff, size #1; Shengmingfaxian Medical Products, Shenzhen, China) was placed and tightly wrapped around the hindlimb proximal to the rat, and a pressure of 300 mmHg was applied to compress the lower‐limb muscle for 3 h. Morphine (2 mg/kg) was administered intraperitoneally as an analgesic, followed by repeat doses every 3 h to maintain analgesia throughout the experiment. For the sham group, all surgical procedures were performed exactly as in the injury groups, except that the compression cuff was not applied and no balloon catheter was inserted into the fascial compartment. The sham animals were maintained under anesthesia for the same duration as the injury groups.

After the removal of the injury‐inducing device, an incision of approximately 2 mm was made in the Achilles tendon of the rat, and the posterior shallow fascial compartment was incised. The red connector of the pressure measuring device was connected to the pressure sensor (PT‐103N, Chengdu Coman Software Co. Ltd.), and the blue connector was connected to an injector containing 10 mL of saline. The injector was pulled back to create a vacuum between the balloon and tube. Immediately thereafter, the balloon catheter was inserted into the superficial fascial compartment. Appropriate saline solution (~0.3 mL) was slowly injected into the balloon to maintain a pressure of 8–10 mmHg, simulating normal ICP. The blue tube was then turned off to complete the ICP adjustment. The injured limb was immobilized with premade plaster to simulate the pathological process of ACS induced by improper immobilization of the lower limb after trauma. A data acquisition system (BL‐420N, Chengdu Coman Software Co. Ltd.) was used to collect and analyze data, including voltage measurements at 60 Hz. After completion of the above procedures, the injury groups were further maintained with plaster immobilization for different durations corresponding to the 0, 1, 2, 3, and 8 h groups, respectively. The 0 h group was euthanized immediately after plaster immobilization. The sham group was maintained under the same conditions and euthanized at 8 h after the sham procedure. After the operation, the rats were placed in a warm, quiet environment, and their activity, diet, and wound conditions were closely observed. No animals died during the experiment. Rats in each time point group (0, 1, 2, 3, and 8 h after injury) were euthanized at their respective designated time points. The overall experimental endpoint for the study was set at 8 h after injury, at which time the rats were euthanized by inhaling an excessive dose of isoflurane (5%, 3 L/min) until respiratory and cardiac arrest were confirmed, and muscle tissue was collected to assess irreversible injury.

To ensure animal welfare, at least one trained investigator remained in the laboratory to continuously observe the animals' general condition and to measure vital signs at irregular intervals throughout the experiment. These measurements were formally recorded every 15 min, but the animals were under near‑continuous visual and physiological monitoring between recordings.

### 2.3 Laser Speckle Imaging

Limb perfusion in both limbs of rats was continuously imaged using the Laser Speckle Contrast Imaging System RFLSI III (RWD, CA, USA). Briefly, the region of interest (ROI) of the limb vessels in both limbs was segmented, and the blood perfusion in the limb region was quantified by measuring the average intensity of the red pixels within the ROI using Image J.

### 2.4 Ischemic Score Analysis Laser Speckle Imaging

A modified ischemia scoring method was defined. Real‐time images of rat limbs after injury were captured by the Laser Speckle Contrast Imaging system, and ischemia scoring was accomplished by visual assessment of paw discoloration.

### 2.5 Histology Staining

The rat tibialis anterior muscle was taken at 0, 1, 2, 3, and 8 h after injury and stained with hematoxylin and eosin (H&E). The animals were euthanized, and the rat tibialis anterior muscles were taken and fixed in 4% paraformaldehyde solution for 24 h. The tissue was then dehydrated in ethanol of varying concentrations, cleared in xylene, and embedded in wax. The embedded tissues were cut into 4–5‐μm‐thick sections, deparaffinized in water, stained with H&E and observed under a microscope.

### 2.6 Inflammatory Factor Detection

Rat serum was collected via the abdominal aorta at 0, 1, 2, 3, and 8 h after injury. The levels of inflammatory factors Interleukin 1β (IL‐1β) and tumor necrosis factor α (TNF‐α) were determined according to the instructions of the enzyme‐linked immunosorbent assay (ELISA) kit. The optical density of each well was measured using an enzyme‐labeled instrument, and the levels of IL‐1β and TNF‐α in rat serum were quantified. Rat TNF‐α ELISA Kit (MM‐0180R1) and Rat IL‐1β ELISA Kit (MM‐0047R1) were purchased from Meimian Industrial Co. Ltd. (Jiangsu, China).

### 2.7 Biochemistry Indicator Measurements

The serum levels of lactose, malondialdehyde (MDA), creatine kinase (CK), and urea nitrogen (BUN) were measured according to the instructions of the biochemistry kits. Lactose, MDA, CK, and BUN kits were purchased from Aidisheng Biotechnology Co. Ltd. (Jiangsu, China). The optical density was measured in a spectrophotometer. Calculate the serum levels of LAC, MDA, CK, and BUN according to the formula provided with the kit.

### 2.8 Statistical Analysis

The data were analyzed using SPSS statistical software. First, the data were tested for normality using the Shapiro–Wilk test to determine whether the data were normally distributed. If the data were normally distributed, one‐way ANOVA was used to analyze the differences between the five groups of samples. If the ANOVA results showed significant differences, the Least Significant Difference or Tukey's test was further used to compare the two groups. If the data did not follow a normal distribution, the nonparametric Kruskal–Wallis rank‐sum test was chosen. Data are presented as mean ± SEM, *p* < 0.05 is considered statistically different.

## 3 Results

### 3.1 Modeling and Study Design

**Figure 1:**
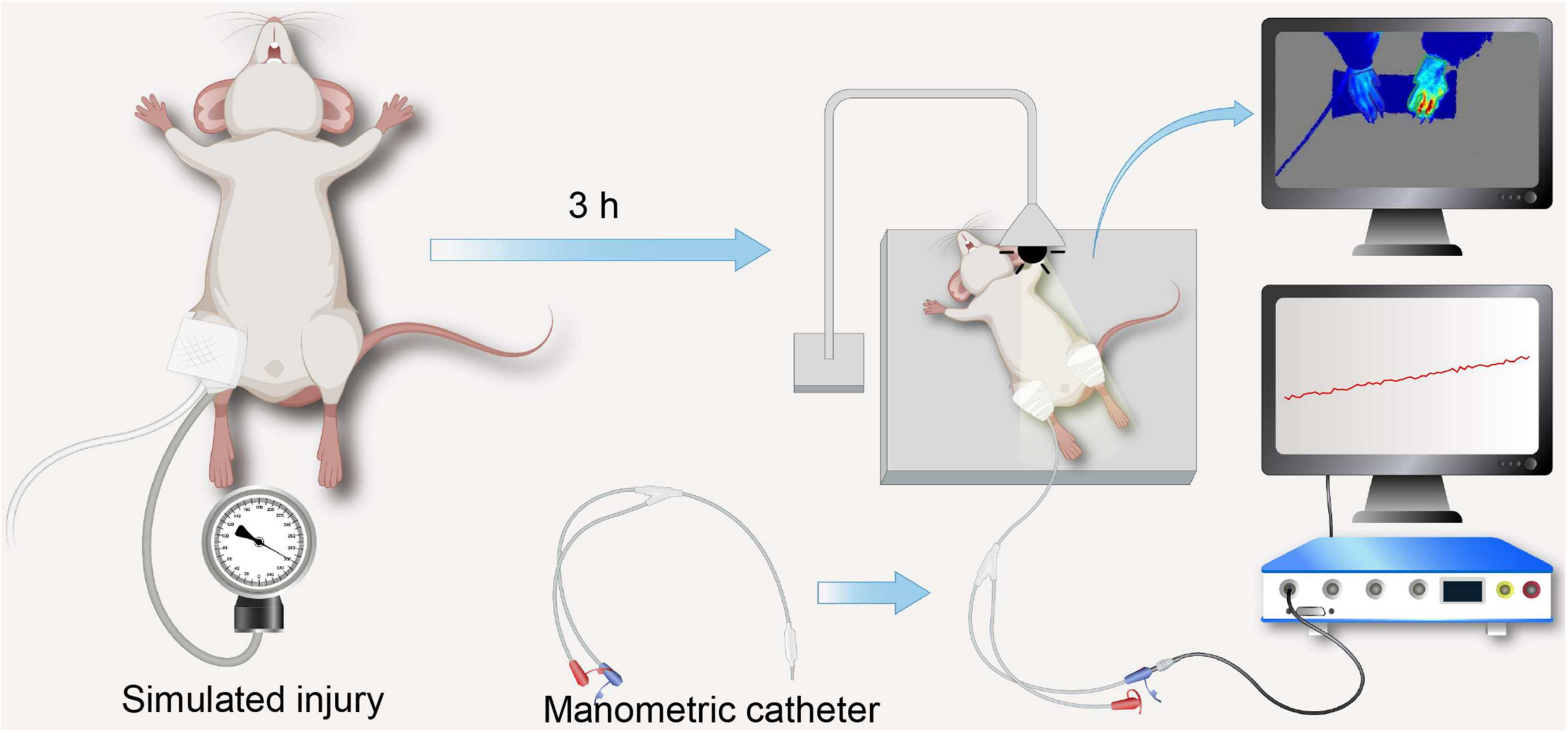
Experimental flow pattern diagram.

The present study was conducted to develop an animal model of ACS and investigate the physiological changes in rats following injury. The experimental design is shown in Figure 1. First, the neonatal blood pressure cuff was attached to the rat's hindlimb proximal to the rat, inflated to about 300 mmHg, and squeezed continuously for 3 h, thereby inducing ACS. After the injury, a 2‐mm incision was made in the Achilles tendon of rats to open the skin and fascia. Subsequently, the adjusted balloon catheter was inserted into the rat's fascia chamber for continuous ICP monitoring. According to the results of the previous pretest, the fluctuation of blood flow and pressure in the hindlimb proximal of rats tends to stabilize after 2 h, so the ICP was recorded continuously for 2 h in this study. At the same time, the rats' hindlimbs were scanned with an imaging device to obtain blood perfusion images.

### 3.2 Pressure Changes During ACS Formation

**Figure 2:**
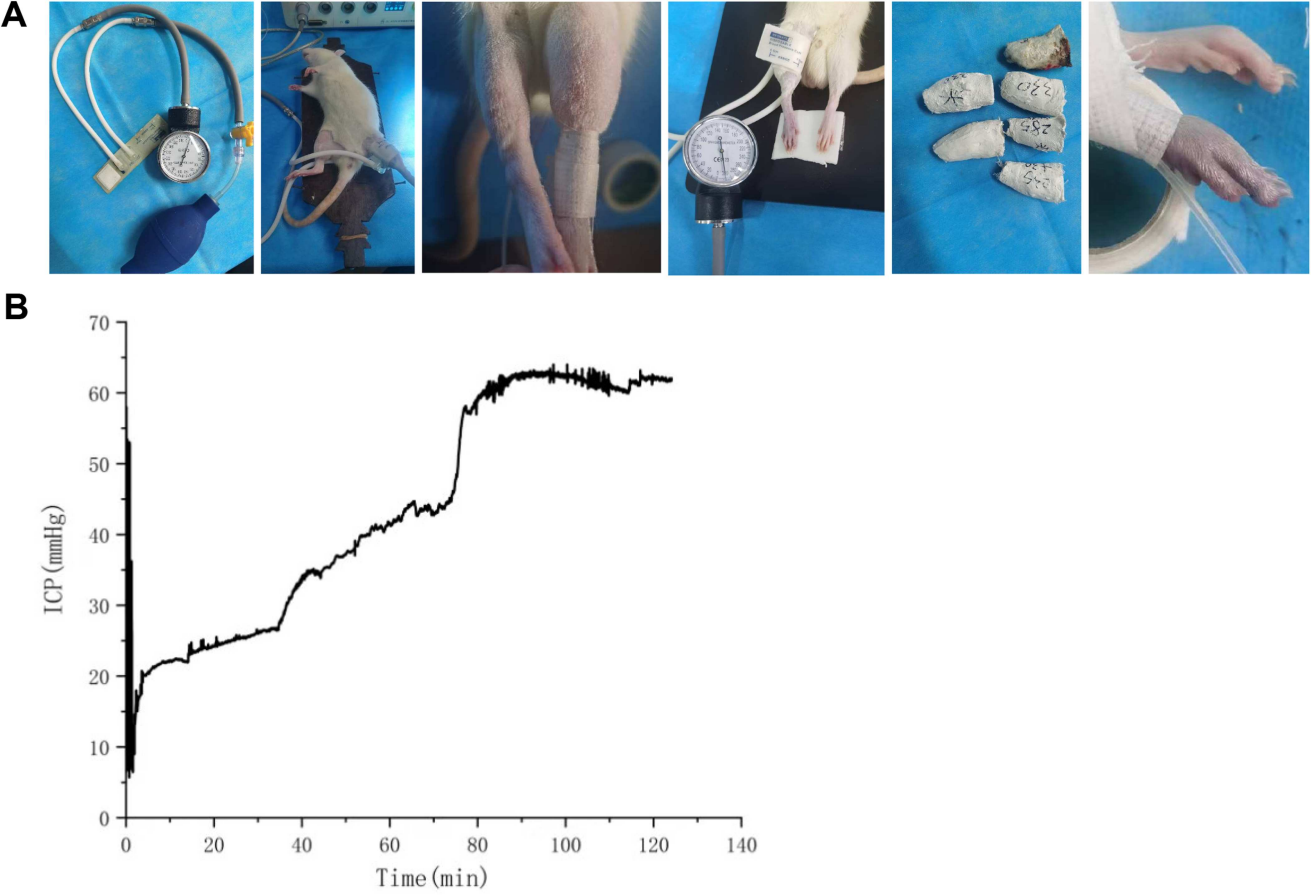
The measurement process and results of the intracompartmental pressure (ICP). (A) Operation and processing of the ACS animal model construction. From left to right: neonatal blood pressure cuff, injury process in rats, swollen hindlimb proximal after injury and immobilized with plaster, balloon manometry catheter, and attaching balloon catheter to hindlimb. (B) Line graph of ICP over time during ACS modeling. The horizontal coordinate is time (min), and the vertical coordinate is ICP (mmHg). ACS, acute compartment syndrome.

Significant swelling of the hindlimb of rats was observed in the ACS group after injury (Figure 2A). And the ICP data showed the dynamic evolution of ICP over time (Figure 2B). As time progressed, the hindlimb ICP increased continuously (0–80 min) and then entered a plateau phase (80–120 min). During this process, ICP increased from baseline to 30 mmHg in about 35 min and to 50 mmHg in about 75 min, meeting the diagnostic criteria for ACS [22, 23]. Notably, the ICP rose sharply, peaking at 60–80 min, consistent with the exponential growth of the pressure–volume curve. When intrafascial swelling reaches a certain level, a small increase in content or muscle swelling may result in a large increase in ICP. These suggested that the model could effectively reproduce the evolution of pressure during ACS development.

### 3.3 Circulatory Hemodynamic Changes in the Hindlimbs

**Figure 3:**
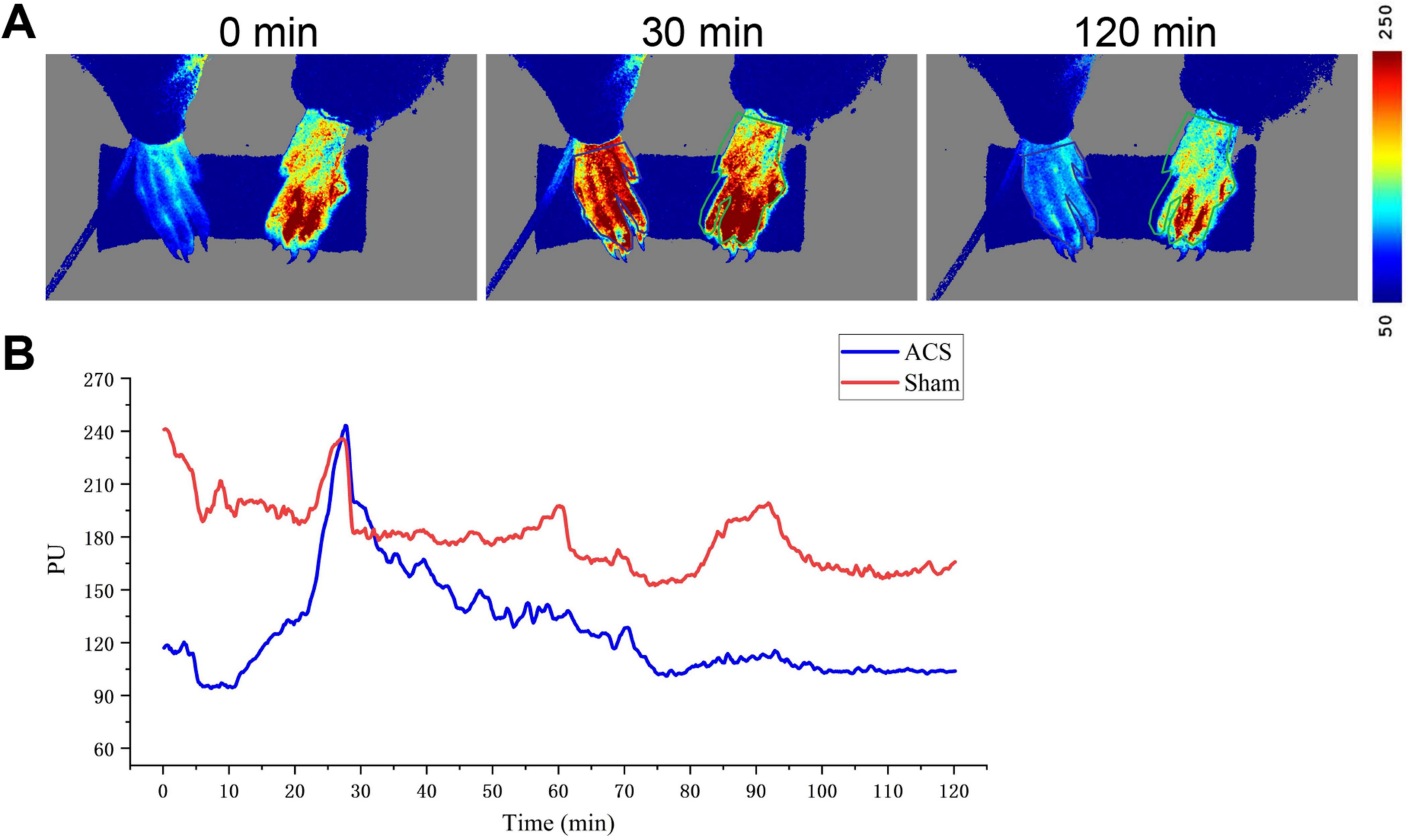
Circulatory perfusion monitoring of the hindlimbs. (A) Representative graph of the thermographic distribution of blood flow in the hindlimbs of rats during 120 min. Left hindlimb, ACS injury; right hindlimb, Sham. The redder colors indicate higher perfusion, and the bluer colors indicate lower perfusion. (B) Line graph comparing the changes in perfusion over time in the ACS and Sham groups during 120 min. The horizontal coordinate is time (min), and the vertical coordinate is perfusion volume (PU). ACS, acute compartment syndrome.

Visual images of blood flow distribution in the hindlimbs were obtained by the imaging system. Blood perfusion in the ACS group showed characteristic three‐step changes (Figure 3A). At the beginning of the injury, the blood perfusion of the ACS group was at about 50% of the basal value (Figure 3B). This was followed by a period of compensatory recovery to the level of the Sham group at about 27 min. However, starting at about 30 min, a progressive decrease in hindlimb perfusion was observed in both the Sham and ACS groups, accompanied by a gradual increase in ICP, which plateaued at 80 ± 3 min. Blood flow‐pressure correlation analysis showed a negative correlation between perfusion and pressure at ICP > 30 mmHg. Notably, this experiment included 24 rats, of which 20 provided valid data. Among these, 16 successfully reached an ICP of 30 mmHg, yielding an 80% success rate, and the remaining four were excluded from the analysis due to data acquisition anomalies. These results suggested that changes in ICP were a key factor affecting hindlimb blood flow, and that increased pressure could decrease hindlimb perfusion.

### 3.4 Pathological Process of Lower Limb Injury in Rats

**Figure 4:**
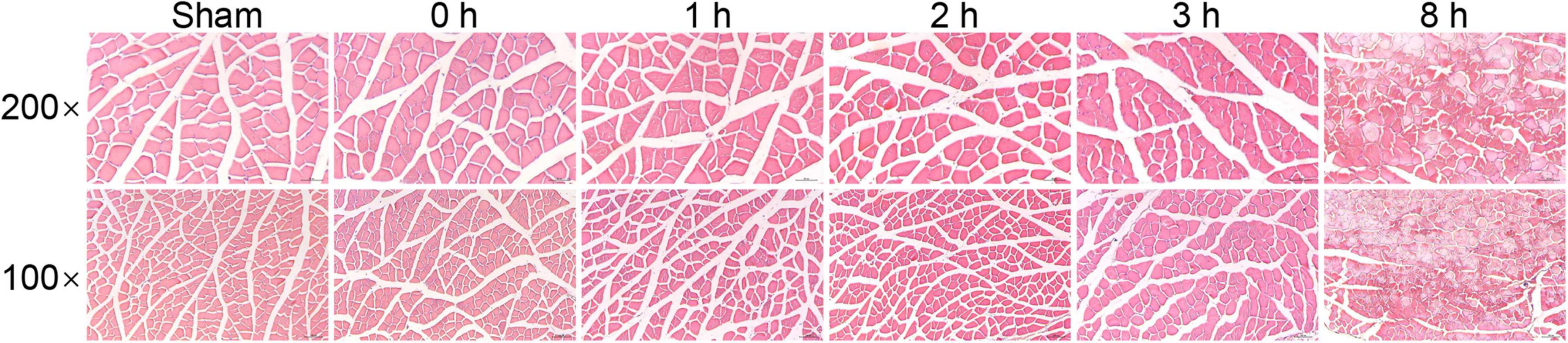
Representative images of hematoxylin and eosin staining of rat hindlimb muscle tissue at different time points after injury (200× and 100×). From left to right: Sham, 0 h after injury, 1 h after injury, 2 h after injury, 3 h after injury, and 8 h after injury.

The muscle tissues at different time points after injury (0, 1, 2, 3, and 8 h) were observed through H&E staining, and typical time‐dependent pathological changes were observed (Figure 4). The muscle fibers of the Sham group were arranged regularly, with clear transverse boundaries, and no abnormalities in the mesenchyme. At 0 h after injury, the tissue structure was normal, and no obvious difference was found in the morphology and arrangement of muscle fibers. After 1 h, some of the muscle fibers began to show mild swelling, while the overall structure remained relatively orderly. The changes in muscle fibers in the 2 h postinjury group were more obvious than those in the 1 h group, with more significant swelling. The abnormal behavior of the muscle fibers in the 3‐h postinjury group was further worsened, with more prominent swelling and disorganization, and inflammatory cell infiltration began to appear. When the injury progressed to 8 h, the muscle fiber structure suffered more serious damage, with phenomena such as muscle fiber rupture and dissolution observed, and the intermuscular space between the muscle fibers was enlarged, and the inflammatory reaction was more intense, indicating that the muscle tissue had been seriously damaged after a long period.

### 3.5 Changes of Injury on Serum Indices in Rats

**Figure 5:**
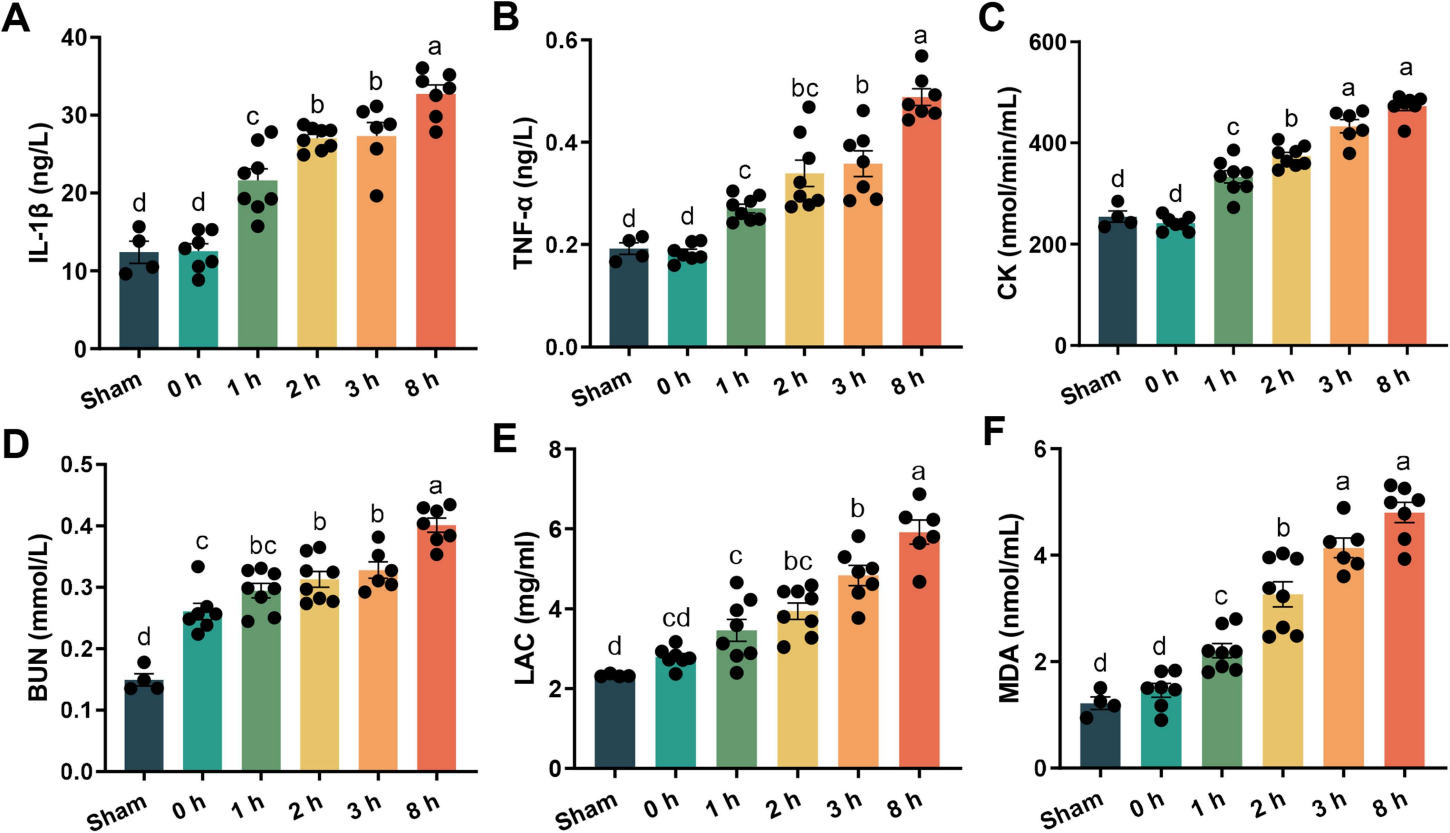
Changes of biochemical indexes in the serum of rats at different time points after injury (0, 1, 2, 3, and 8 h). (A, B) Inflammatory factors IL‐1β and TNF‐α. (C) Muscle injury index CK. (D) Kidney function index BUN. (E) Anaerobic metabolism index LAC. (F) Lipid peroxidation index MDA. Black scattered dots are specific data points, and letters on the bars indicate statistically significant differences (*p* < 0.05). BUN, urea nitrogen; CK, creatine kinase; IL‐1β, interleukin 1β; LAC, lactate; MDA, malondialdehyde; TNF‐α, tumor necrosis factor α.

To further evaluate the persistent damage caused by blood pressure cuff‐induced injury in rats, we examined the biochemical changes related to ACS formation at 0, 1, 2, 3, and 8 h postinjury. The results showed that the proinflammatory cytokines IL‐1β and TNF‐α remained at low levels in the Sham group, similar to 0 h after the injury group (Figure 5A,B). As time passed, they increased and reached their highest value at 8 h, indicating that the inflammatory response was progressively enhanced after injury. The content of CK, an indicator of muscle injury, was relatively low in the Sham group (Figure 5C). It gradually increased from 0 h and was significantly higher at 8 h than at the other time points, suggesting that the degree of muscle damage aggravated over time. The expressions of renal function indicators BUN, the anaerobic metabolic product LAC, and the lipid peroxidation product MDA also continuously increase over time, indicating that the degree of injury is gradually worsening (Figure 5D–F).

## 4 Discussion

As a common emergency in the field of trauma, ACS has a high incidence of fractures and crush injuries [3]. Its pathological feature is that pressure within the confined fascial chamber increases dramatically, triggering a cascade of microcirculatory disorders and tissue ischemia [22]. Due to the hidden symptoms in the early stage, the diagnosis is easily delayed. If not intervened in time, ACS will not only block the blood circulation of the limbs, leading to muscle necrosis and even amputation, but also induce rhabdomyolysis, which will lead to acute renal failure and systemic inflammatory response syndrome, seriously threatening patients' lives [1, 24, 25]. Once ACS is diagnosed, immediate action should be taken to reduce the ICP and restore blood circulation [22]. Although fasciotomy and decompression are the main treatments for ACS, it is not suitable for all patients, and risks such as infection and poor wound healing exist in the postoperative period [26, 27]. In recent years, some scholars have also believed that ACS treatments were too aggressive. Most patients do not need surgical incision and decompression, and ICP can be reduced spontaneously, especially in patients with blisters after limb injuries, but they suffer from huge surgeries, prolonged hospitalization, and high medical costs [28, 29]. Therefore, the construction of experimental models that can accurately simulate the pathogenesis of ACS is essential for early diagnosis and the development of more effective therapeutic strategies.

Current modeling and manometry methods have many limitations in ACS research. In animal models, the anatomical and physiological differences among rats, rabbits, and human fascial compartments render the simulated ACS process inconsistent with reality [27]. Especially, rats are the most frequently used experimental subjects due to their small size and low cost, suitable for large‐scale experiments [16]. However, the rat fascia has excessive elasticity and is prone to significant swelling, and the elevation of ICP and hindlimb ischemia are not obvious [13]. Our pretest found that rats without plaster after injury induction showed no significant fluctuation in ICP, slight cellular swelling, and that the injury subsided on its own after about 3 days without further injury. Therefore, in the present study, we applied plaster to limit the expansion of rat fascia, thereby more realistically simulating the role of limited fascial expansion in the progression of ACS. Currently, the most used method to construct the model is the intrafascial fluid infusion method, which centers on a continuous fluid drip through the intrafascial compartment to simulate the high ICP pathology after trauma [27]. This method has obvious limitations, including the difficulty of maintaining a stable pressure, and the uninterrupted drip and the systemic reaction triggered by fluid absorption, which can hardly be ignored in the results. Alternatively, the use of a catheterized balloon placed within the fascial compartment or an inflatable cuff around the limb to simulate the clinical state of high ICP is also a classic modeling method [19]. In practice, however, the placement of a balloon within the fascial compartment disrupts the fascial structure and may introduce additional inflammation or edema. The inflatable cuff surround makes it difficult to simulate the pressure gradient distribution of ICP due to bleeding or edema in clinical ACS, and the mechanical artificial pressure only simulates the result of elevated pressure, ignoring important pathological mechanisms such as posttraumatic systemic inflammatory response and coagulation changes. Some studies have attempted to elevate the ICP with fracture followed by crush to form the ACS [27]. However, our pre‐experiment revealed that fracture trauma tends to disrupt the integrity of the fascial compartment. Although it is clinically accepted that fascial integrity does not affect ICP, the effect was significant in the rats, leading to poor reproducibility [30]. Another study simulated ACS by blast‐induced fracture, which required surgical suture after fascial compartment disruption, and then built a high‐pressure environment with a high impact of manual intervention. Moreover, this study measured only pressure and lacked blood indices and pathology to provide strong evidence of secondary damage [13]. Compared with other studies, the present study used direct crush injury to develop a model that more closely matched the pathogenesis of clinical fractures. With the gradual increase in intrafascial pressure, the muscle tissue showed initial signs of edema and ischemia, as well as serious lesions such as swelling and fracture of muscle fibers, which were highly consistent with the biopsy results from clinical tissues [31, 32]. Meanwhile, with the self‐developed balloon catheter, high‐frequency continuous pressure measurement of intrafascial pressure can be realized after one time of zero adjustment, which avoids the interference of local pressure and inflammatory manifestations caused by the traditional methods, such as repeated injection of fluids, and is able to accurately capture the details of pressure changes [27, 33, 34]. In addition, the modeling process in this study is greatly simplified, eliminating reliance on complex surgery and specialized equipment, reducing experimental cost and difficulty, and improving reproducibility and stability. Notably, our choice of injury method and the use of plaster immobilization were not arbitrary but were informed by multiple pre‐experiments and existing research evidence. In our pre‐experiments, simple crush injury, bone injury, or ischemia‐only intervention (without plaster immobilization) induced only mild hindlimb swelling in rats, with no significant fluctuations in ICP that met the diagnostic criteria for ACS. The fundamental reason for this phenomenon is that the fascial extensibility of rat hindlimbs is far higher than that of human lower extremities, which is a well‐recognized species‐specific limitation of rat ACS models. Thus, plaster immobilization was applied in our model to simulate the low‐extensibility fascial compartment structure of humans, which is a necessary design to obtain a clinically relevant ACS model with measurable and diagnostic ICP elevation.

In our model, pressure and blood flow in the rat hindlimb fascial cavity exhibited a distinct pattern of change following simulated injury. The pressure reached its maximum value in about 80 min, and the blood flow started to decrease after returning to the level of the Sham group in about 30 min. The phenomenon suggested that the decrease in blood flow depends not only on the peak pressure but also on vascular status and local inflammatory manifestations [20, 35]. During the rise of ICP, blood vessels were gradually compressed, and blood flow was gradually reduced by the peripheral inflammatory reaction and changes in the state of the vessels themselves. Our pretest data showed that the ICP could be maintained at 60 mmHg with little change over 2–8 h after injury, and the perfusion volume did not change much, so we cut off the measurement time for ICP and blood flow at 2 h. We chose 8 h as the cutoff time for the overall observation because muscle necrosis typically appears after 6 h of ischemia. Continued observation for 6 h after ACS formation, that is, until 8 h postinjury, revealed that pathological and hematologic findings were more severe at this stage than before, confirming that elevated ICP and decreased blood flow after ACS formation can lead to further secondary damage. In contrast to other studies, the ICP trend in this model was similar to that observed in clinical ACS, with a rapid pressure‐increase phase and a plateau phase [16, 36]. Most studies have also observed a gradual decrease in blood flow as ICP increases. However, the present study clarified the three‐step changes in perfusion and the quantitative pressure‐blood flow relationship, reflecting the body's initial compensatory mechanism and subsequent compensatory failure. A significant decrease in early blood perfusion was observed in a similar trauma model, corresponding to a decrease in blood perfusion at the initial stage of injury in the present study, but which did not detail the subsequent recovery [37]. We found that compensatory recovery of blood perfusion occurred in the ACS group at approximately 27 min, likely because the body sensed ischemic and hypoxic signals and initiated vasodilation and collateral circulation opening to maintain blood supply [38]. From about 30 min onwards, limb perfusion gradually decreased as ICP rose, and perfusion entered a plateau after about 80 min. These suggested that elevated ICP had a continuous effect on limb perfusion and that raising ICP to a certain level would break the body's compensatory mechanisms. The combination of blood flow and ICP showed perfusion was negatively correlated with ICP when ICP was > 30 mmHg, consistent with previous findings and confirmed that a change in ICP was a key factor affecting hindlimb blood flow [14]. These results demonstrated that an increase in ICP beyond the threshold would decrease perfusion and trigger ischemia and hypoxia‐induced injury in muscle tissue, suggesting that clinical treatment requires accurate monitoring of ICP and providing new avenues for exploring the pathological mechanisms and therapeutic strategies of ACS.

To explore the pathology and changes of muscle necrosis, the ACS model was analyzed with H&E staining and biochemical indexes. The results showed that the histopathological changes were obvious at all time points after injury. From basically normal at 0 h after injury, to mild swelling of some muscle fibers at 1 h, increasing swelling at 2 h, increasing swelling, disordered alignment, and inflammatory cell infiltration at 3 h, and finally to severe destruction of muscle fibers and a strong inflammatory reaction at 8 h. Compared with other research models, this process is clearer and more gradual, and is closer to the pathologic changes in muscle tissue of clinical patients [27]. The proinflammatory cytokines IL‐1β and TNF‐α, muscle damage index CK, renal function index BUN, and anaerobic metabolites LAC and lipid peroxidation products MDA increased over time after injury, following a typical time‐dependent pattern, which was highly compatible with the results of continuous ICP and blood flow tracking. In the first 3 h, no significant secondary damage was observed due to the short duration of ischemia. In the 8 h, there was a significant positive result, and the index change was consistent with the degree of inflammatory reaction and muscle damage on H&E staining. Compared with other studies, the magnitude and timing of the index change in this model were more specific, and it could more accurately and reliably reflect the biochemical and pathological changes of ACS and provide a quantitative basis for assessing disease progression [14, 39].

Despite the promising features of our novel ACS model, several limitations should be acknowledged. First, due to the inherent anatomical differences between rodent and human hindlimbs—particularly the higher extensibility of rat fascia—we applied a plaster cast to simulate the low‑compliant human fascial compartment. While this manipulation was necessary to achieve clinically relevant ICP elevation, it introduces an external constraint that may not fully replicate human ACS pathophysiology. Second, our model focused on the early phase of ACS (within 8 h postinjury) to investigate the window for potential interventions; consequently, long‑term outcomes such as neuromuscular dysfunction, fibrosis, and functional recovery were not assessed. Finally, functional outcome measures (e.g., pain behavior and motor function) were not included, which limits the model's clinical relevance. Future studies using larger animals, longer observation periods, more specific biomarkers, and functional assessments are warranted to further validate and refine this model.

## 5 Conclusion

As a technically elegant and low‐cost preclinical model with a novel continuous ICP monitoring device, the ACS injury model and pressure measurement device developed in this study have significant advantages in simulation realism, operational simplicity, and data integrity. The comprehensive analysis of pressure, blood flow, tissue morphology, and biochemical indexes in the model further revealed the pathophysiological mechanism of ACS, and provided new ideas and methods for the early diagnosis and optimization of treatment strategies for ACS.

## Author Contributions

Conception: Sishu Guan and Lianyang Zhang. Data curation: Xingchen Lu, Chang Liu, Yang Li, and Hui Zhao. Formal analysis: Sishu Guan and Yi Zhang. Methodology: Sishu Guan and Lianyang Zhang. Validation: Xingchen Lu, Chang Liu, Yi Zhang, and Hui Zhao. Writing – original draft: Sishu Guan and Xingchen Lu. Writing – review and editing: Lianyang Zhang, Chang Liu, Yi Zhang, Hui Zhao, and Yang Li. Final approval of manuscript: All authors.

## Funding

The authors have nothing to report.

## Ethics Statement

All animal protocols of handling and treatment were reviewed and approved by the Laboratory Animal Welfare and Ethics Committee of the Army Medical University (Approval No. SYXK (YU) 2022‐0018).

## Consent

The authors have nothing to report.

## Conflicts of Interest

The authors declare no conflicts of interest.

## Data Availability

The data that support the findings of this study are available from the corresponding author upon reasonable request.
